# Brain glycogenolysis, adrenoceptors, pyruvate carboxylase, Na^+^,K^+^-ATPase and Marie E. Gibbs' pioneering learning studies

**DOI:** 10.3389/fnint.2013.00020

**Published:** 2013-04-03

**Authors:** Leif Hertz, Junnan Xu, Dan Song, Ting Du, Enzhi Yan, Liang Peng

**Affiliations:** Department of Clinical Pharmacology, China Medical UniversityShenyang, People's Republic of China

**Keywords:** astrocyte, glutamate, glycogen, learning, slow neuronal afterhyperpolarization

## Abstract

The involvement of glycogenolysis, occurring in astrocytes but not in neurons, in learning is undisputed (Duran et al., 2013). According to one school of thought the role of astrocytes for learning is restricted to supply of substrate for neuronal oxidative metabolism. The present “perspective” suggests a more comprehensive and complex role, made possible by lack of glycogen degradation, unless specifically induced by either (1) activation of astrocytic receptors, perhaps especially β-adrenergic or (2) even small increases in extracellular K^+^ concentration above its normal resting level. It discusses (1) the known importance of glycogenolysis for glutamate formation, requiring pyruvate carboxylation; (2) the established role of K^+^-stimulated glycogenolysis for K^+^ uptake in cultured astrocytes, which probably indicates that astrocytes are an integral part of cellular K^+^ homeostasis in the brain *in vivo*; and (3) the plausible role of transmitter-induced glycogenolysis, stimulating Na^+^,K^+^-ATPase/NKCC1 activity and thereby contributing both to the post-excitatory undershoot in extracellular K^+^ concentration and the memory-enhancing effect of transmitter-mediated reduction of slow neuronal afterhyperpolarization (sAHP).

Gold and Korol (2012) discussed a key role for glycogenolysis in memory establishment and reviewed evidence that moderate levels of systemically released adrenaline enhance and higher levels impair learning. They concluded that memory-enhancing effects mainly reflected increases in blood glucose levels, supporting brain processes engaged during memory establishment. It is well known that glucose and other substrates like short-medium chain fatty acids may improve memory, even under pathological conditions (Watson and Craft, 2004; Henderson and Poirier, 2011).

The intentions of this perspective article are (1) to more strongly emphasize that the glycogenolytic effect in brain is caused by noradrenaline released within the central nervous system and to explain why the stimulation is not restricted to neurons, (2) to emphasize that a need for glycogenolysis during the establishment of memory was first shown by Marie E. Gibbs' pioneering studies, which were overlooked in the Gold/Korol paper, (3) to elaborate on additional recent observations regarding K^+^ uptake in astrocytes, which support the importance of astrocytic glycogenolysis during learning and are consistent with early concepts by Marie Gibbs and her colleagues, and (4) to refer to an additional Gibbs paper, showing that brain injection of not only lactate but also octanoate or β-hydroxybutyrate, but *not* glucose, can rescue learning disability induced by previous administration of oligomeric β-amyloid (Aβ) 1–42.

With few exceptions, systemically released adrenaline does *not* enter the brain (Weil-Malherbe et al., 1959). Small amounts of adrenaline, together with larger amounts of the slightly different molecule, noradrenaline, are produced in *locus coeruleus* and a few additional neuronal nuclei in the brain stem, from which noradrenergic nerve fibers reach the entire central nervous system (reviewed by Hertz et al., 2004). Transmitter release both in genuine synapses and from non-synaptic axonal release sites (Beaudet and Descarries, 1978), secures that noradrenaline effects can be exerted on all brain cell types (O'Donnell et al., 2012). This includes astrocytes (Bekar et al., 2008), where glycogenolysis is a key target (Hertz et al., 2010). Recently, glycogen's importance for learning has been substantiated by Duran et al. (2013), demonstrating severe disturbances in long-term memory formation *and* learning-dependent synaptic plasticity in mice lacking brain glycogen synthase.

One group of authors regards transfer to neurons of glycogen-derived lactate as the main reason for the memory-enhancing effect of glycogenolysis. Suzuki et al. (2011) showed that extracellular lactate levels in rat hippocampus increase during learning, and that this increase, memory and long-term potentiation (LTP) were abolished by glycogenolytic inhibitors. Disruption of the expression of astrocytic lactate transporters also caused amnesia and LTP impairment, which could be rescued by L-lactate, but not by glucose. Specific knock-down of the neuronal monocarboxylate transporter MCT2 led to amnesia, against which neither L-lactate nor glucose could protect. These findings are consistent with a proposed concept, the astrocyte-to-neuron lactate shuttle (ANLS), that lactate release from astrocytes and its uptake in neurons might be important for brain function (Pellerin and Magistretti, 2012). Newman et al. (2011) confirmed memory impairment by inhibition of glycogenolysis. Again, the impairment could be counteracted by either lactate or glucose, and blockade of the neuronal monocarboxylate transporter impaired memory, with no reversal by either lactate or *glucose*. Since the ability of neurons to metabolize glucose should be intact, the authors further advanced the concept of astrocyte-to-neuron transfer of lactate by concluding that lactate might be a *specially important substrate* for neurons during working memory by rapidly providing additional energy. Consistent with this concept Dringen et al. (1993) had shown in cultured astrocytes that glycogenolysis causes release of lactate, not of glucose, and that glycogen is continuously re-synthesized in the presence of glucose. However, the rate of glycogen turnover *in the living brain* is very modest compared to that of glucose breakdown (Öz et al., 2012). Furthermore, although the observed learning deficits reported by both Suzuki et al. (2011) and Newman et al. (2011) are solid, it can not be excluded that inactivation of transporters may have additional effects. Effects by alpha-cyano-4-hydroxycinnamate (4-CIN) may include inhibition of mitochondrial entry of pyruvate (McKenna et al., 2001; Rae et al., 2012), and MCT2 knock-down might have a similar effect (Hashimoto et al., 2008). Also, although an Alzheimer study by Gibbs et al. (2009) showed that lactate, octanoate, and β-hydroxybutyrate could rescue Aβ-impaired memory, the authors concluded that this was due to an effect on astrocytic metabolism.

Many studies by Marie E. Gibbs describe the importance of noradrenaline-stimulated glycogenolysis for one-trial aversive learning in the day-old chick, a precocious animal. The first of these resembles the papers discussed above in emphasizing metabolic aspects (O'Dowd et al., 1994). It demonstrates dependence on iodoacetate-inhibitable glycolysis, regardless whether glucose or glycogen is metabolized, but it also shows time periods with decreased glycogen content in the brain. Later, attention was drawn toward the importance of glycogenolysis for glutamate synthesis (Figure 1), in studies culminating in the demonstrations (Gibbs et al., 2006, 2007) that (1) administration of the glycogenolytic inhibitor DAB prevented a normally observed rise in cellular glutamate level soon after training, (2) no compensatory decrease occurred in contents of other amino acid interacting with the tricarboxylic acid (TCA) cycle, indicating *de novo* synthesis of glutamate (or inhibited breakdown), and (3) learning was inhibited by intracranial injection of DAB only during highly specific time periods. These included periods when glycogen content was decreased, but also a time period around 30 min post-training, when no such decrease could be shown. The importance of glycogen to support glutamatergic transmission has also been shown in a cell culture study by different authors (Sickmann et al., 2009).

**Figure 1:**
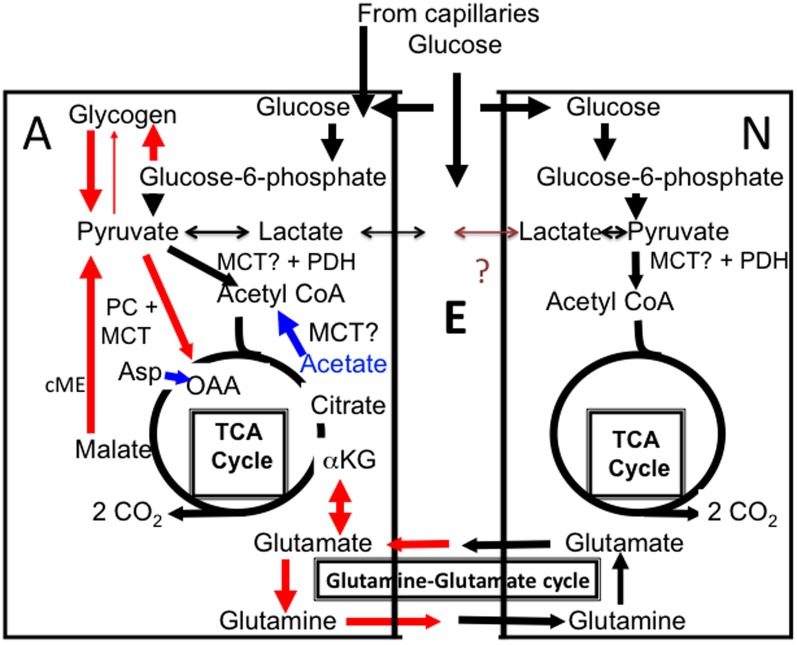
**Cartoon of glucose metabolism in astrocytes (A, left) and neurons (N, right), with extracellular space shown as E.** Glucose is accumulated both in neurons and astrocytes and metabolized via glucose-6-phosphate to pyruvate and in astrocytes also used for glycogen synthesis. Pyruvate is mainly metabolized via pyruvate dehydrogenase (PDH) to acetyl coenzyme A (acetyl CoA) that, in the mitochondrial tricarboxylic acid (TCA) cycle, condenses with oxaloacetate (OAA) to form citrate (pathway shown in black). Lactate can also be formed from pyruvate. Some of this lactate may be released from the cell, although glycogen-derived lactate may be formed and released slowly *in vivo*. According to some of the authors discussed in the present “perspective” release of glycogen-derived lactate may be followed by its uptake into neurons and use as an important metabolic substrate for neurons during learning. The postulated net flux from astrocytes to neurons has never been proven and a brown question mark is shown in the Figure. Citrate is further metabolized via α-ketoglutarate (α-KG) to eventually regenerate oxaloacetate, which condenses with another molecule of acetyl CoA, and so on. During the process two molecules of CO_2_ are released. The purpose of this cycle is solely generation of ATP. In astrocytes additional pathways are also operating (red), which is a major reason for the importance of astrocytes, including astrocytic glycogenolysis. Glucose is used for synthesis of glycogen, which is metabolized to glucose-6-phosphate (by a different route than glucose and therefore selectively inhibitable by DAB), and from there by the same pathway as glucose, to pyruvate. Pyruvate can be carboxylated to form a “new” molecule of oxaloacetate, which condenses with acetyl CoA to form a “new” molecule of citrate, from which α-ketoglutarate and glutamate can be formed and transferred to neurons via glutamine release (red). Glutamate released as a transmitter to the extracellular space is virtually quantitatively taken up by astrocytes. Eighty-five percent of the accumulated amount is converted to glutamine by the astrocyte-specific glutamine synthetase and carried back to neurons in the “glutamine–glutamate (Gln–Glu) cycle” where it can be re-utilized as transmitter glutamate or converted to γ-aminobutyric acid (GABA). The remaining 15% is degraded in astrocytes after conversion by cytosolic malic enzyme (cME) to α-ketoglutarate, then to malate, which exits the cycle and is decarboxylated to pyruvate. This pyruvate can then be oxidized in the TCA cycle via acetyl CoA, and astrocytic formation of glutamate combined with subsequent glutamate degradation supplies almost as much energy (ATP) as degradation via PDH. Noradrenergic agonists subtype-specifically stimulate glycogen synthesis (via α_2_-adrenergic receptors) and glycogenolysis (via β-adrenergic receptors), as well as pyruvate carboxylase activity (via α_2_-adrenergic receptors). Elevated extracellular concentrations of K^+^ also stimulate both glycogenolysis and pyruvate carboxylation. Lactate can function as a pyruvate precursor or product in both astrocytes and neurons, whereas acetate is an astrocyte-specific precursor of acetyl CoA, but not a precursor for pyruvate carboxylation. However, if both acetate and aspartate (Asp) are supplied (indicated in blue), oxaloacetate, formed from aspartate, and acetyl CoA, formed from acetate, can sustain net production of citrate, α-ketoglutarate and glutamate. Entry of pyruvate or acetate into the mitochondrial TCA cycle requires a transporter, here simply indicated as MCT.

Both human and rodent brain astrocytes *in vivo* oxidatively degrade glucose at a rate, which per cell volume is at least as high as in neurons (reviewed by Hertz, 2011). More than one half of glucose oxidation in astrocytes occurs via *formation and degradation* of glutamate in a complex process, which includes neuronal glutamate uptake and subsequent release, followed by a mainly astrocytic uptake (Danbolt, 2001) and intense glutamate metabolism in astrocytes (Bauer et al., 2012; McKenna, 2012). Figure 1 shows that glutamate synthesis requires pyruvate entry into the astrocytic TCA cycle via two pathways, (1) the conventional pyruvate dehydrogenase-mediated pathway (black in the Figure) and (2) pyruvate carboxylation, catalyzed by pyruvate carboxylase (red in the Figure). This enzyme is not expressed in neurons, but is abundant in astrocytes (Shank et al., 1985; Hutson et al., 2008), where glutamate production is rapid (Yu et al., 1983). Most generated glutamate is transferred via glutamine to neurons and used as precursor for transmitter glutamate and GABA (Figure 1). In the brain *in vivo* the rate of this transfer equals the rate of glucose utilization, i.e., it is extremely fast (reviewed by Hyder and Rothman, 2012). Although about 85% of the flow of glutamine from astrocytes to neurons is compensated for by a flow in the opposite direction of glutamate and some GABA, 15% is not but depends upon continuous glutamate production in astrocytes. Over time the transfer of newly synthesized glutamate equals the subsequent metabolic degradation of GABA and glutamate, to a large extent in astrocytes (Hertz, 2011; McKenna, 2012), However, fluctuations occurring in tissue glutamate levels during brain function (Mangia et al., 2012) must indicate altered glutamate production and/or degradation rates. Established rates of glycogenolysis (see above) are not high enough to enable glycogen to supply the pyruvate carboxylase-dependent one half of the glutamate molecule. Rather, it is required for the function of the pyruvate carboxylase, a question that will be re-discussed toward the end of this communication.

As in the papers by Suzuki et al. (2011) and Newman et al. (2011) memory could in the Gibbs studies be rescued after DAB administration by lactate and by glucose, but only during precisely limited time intervals (tabulated in Gibbs et al., 2008). An amnestic effect of DAB administration 30 sec after training was prevented by glucose administration immediately after training or by lactate injection either immediately after training or anytime during the period 10–20 min post-training. The DAB challenges with glucose/lactate provide *per se* no information about the cell type(s) in which the supplied substrates were metabolized. Acetate, which is generally considered *an astrocyte-specific metabolic substrate* (Muir et al., 1986; Waniewski and Martin, 1998; but see also Rae et al., 2012), could rescue at the same times as lactate. However, this was only the case, when aspartate (which alone had no effect) was administered together with acetate. As can be seen from Figure 1 (blue), such co-administration enables glutamate formation even when pyruvate carboxylation is inhibited, since aspartate is a precursor for oxaloacetate, and oxaloacetate is the initial glutamate precursor generated by pyruvate carboxylation. Consistent with this explanation, administration of glutamine itself at similar time also rescued memory (Gibbs et al., 2008).

Glycogenolysis occurs in response to stimulation of the β-adrenergic receptor, as a β_2_-adrenergic effect in the day-old chick (Gibbs et al., 2008) and a β_1_-adrenergic effect in human brain (Quach et al., 1988). The subtype difference reflects species differences in β-adrenergic subtype expression in fowl and mammals (Fernández-López et al., 1997). The glycogen molecule is unique in being stable under control conditions, but rapidly degraded in response to certain transmitters (Magistretti, 1988), and to even very small increases in extracellular K^+^ concentrations from their resting level (Hof et al., 1988).

Besides responding to β-adrenergic receptor activation (Quach et al., 1988; Subbarao and Hertz, 1990; Gibbs et al., 2008), glycogenolysis is also evoked by some other transmitters, including serotonin (Quach et al., 1982; Magistretti, 1988; Kong et al., 2002; Darvesh and Gudelsky, 2003). In mature cultured astrocytes stimulation of β_1_-adrenoceptors activates protein kinase A (via G_s_) *and* causes an in increase in free cytosolic calcium concentration ([Ca^2+^]_i_) after a G_s_-G_i_ switch (Du et al., 2010) with the latter probably responsible for most of the glycogenolytic effect. Addition of 5 mM extracellular K^+^ concentrations also increases [Ca^2+^]_i_ in astrocytes, secondary to an Na^+^,K^+^-ATPase effect (Xu et al., 2013). A different possibility for glycogenolytic stimulation (Choi et al., 2012) is activation of astrocytic soluble adenylate cyclase (sAC) by depolarization-induced entry of bicarbonate [carried by a Na^+^-bicarbonate cotransporter (NBC)], which increases cAMP (and thus activates protein kinase A), but the cAMP effect is minuscule. Glycogen synthesis is essential for continued glycogenolysis. In astrocytes this process is *also* stimulated by noradrenaline, acting on a different subtype receptor, the α_2_-adrenoceptor (Hertz et al., 2007; Hutchinson et al., 2008). The α_2_-adrenergic receptor is *at least* as densely expressed in astrocytes (per mg mRNA) as in freshly isolated neurons (Hertz et al., 2010). The importance for learning of a continuous glycogen synthesis/glycogenolysis process by temporally selective, interspersed periods of β-adrenergic and α_2_-adrenergic activity has recently been clearly demonstrated by Gibbs and Hutchinson (2012). A distinct decrease of glycogen content in brain immediately after learning, but not around 30 min (Gibbs et al., 2007), in spite of the ability of DAB to inhibit at both times, may also reflect that serotonin acts as the major glycogenolysis-inducing transmitter right after training, whereas noradrenaline does so at 30 min (Gibbs et al., 2008). Serotonin is not known to stimulate glycogen synthesis.

A seminal paper by DiNuzzo et al. (2012) suggested that glycogenolysis may be required for astrocytic contributions to clearance of excess extracellular K^+^ resulting from neuronal K^+^ release during excitation. This suggestion greatly expanded the potential role of glycogenolysis in memory formation. Unless excessively high extracellular K^+^ concentrations are reached, clearance of elevated extracellular K^+^ occurs by activation of Na^+^,K^+^-ATPases. In both astrocytes and neurons Na^+^,K^+^-ATPase activity is regulated in conventional manners by ion sensitivities, i.e., at its intracellular site by Na^+^ stimulation and at its extracellular site by K^+^ stimulation. Due to enzyme affinities, determined by subtype expression levels of its α and β subunits and of the auxiliary protein FXYD (Delprat et al., 2006), only the astrocytic enzyme is stimulated by increases in extracellular K^+^ concentration beyond its normal resting level (Grisar et al., 1980; Hajek et al., 1996). The astrocytic contribution to clearance of elevated extracellular K^+^ seems to be essential for K^+^ homeostasis in brain (Walz, 2000; Somjen et al., 2008; MacAulay and Zeuthen, 2012; Wang et al., 2012). However, in contrast to the excitation-induced increase in intracellular Na^+^ in neurons, no Na^+^ enters astrocytes *spontaneously*, but Xu et al. (2013) showed in cultured astrocytes that slightly elevated extracellular K^+^ activates a glycogenolysis-dependent Na^+^,K^+^-ATPase/endogenous ouabain signaling pathway leading to astrocytic Na^+^ entry. Without this activation elevated K^+^ concentrations are unable to stimulate the astrocytic Na^+^,K^+^-ATPase. Activation of glycogenolysis by elevated extracellular K^+^ concentration allows initial K^+^ reaccumulation in astrocytes, followed by K^+^ release through Kir1.4 channels and neuronal K^+^ uptake when the stimulation of the astrocytic uptake ceases after K^+^ concentration normalization. A role of this process in learning is likely but not yet established.

The astrocytic Na^+^,K^+^-ATPase is also specifically stimulated by isoproterenol, a non-subtype-specific β-adrenergic agonist (Hajek et al., 1996). The neuronal enzyme is stimulated by noradrenaline but not by isoproterenol, and in both cell types the stimulation is inhibited by increased extracellular K^+^ concentrations (Hajek et al., 1996). Although neuronal α_2_-adrenergic stimulation may also be important for memory-enhancing effects by α_2_-adrenergic stimulation (Caetano et al., 2012), β-adrenergic effects are essential (Oh et al., 2009). At *non-elevated extracellular* K^+^ *concentration* β_1_-adrenergic stimulation enhances regulatory volume increase in mouse astrocytes during hypertonic stimulation, again via a glycogenolytic effect (Xu, Song, Du, Yan, Hertz and Peng, unpublished results). The β_1_-adrenergic stimulation of volume increase relies on Na^+^,K^+^-ATPase-dependent stimulation of a combined uptake of H_2_O, Na^+^, K^+^ and 2Cl^−^, mediated by the co-transporter NKCC1 (Hamann et al., 2010). In brain slices extracellular hypertonicity reduces excitatory activity (Huang and Somjen, 1997), an effect similar to that of slow neuronal afterhyperpolarization (sAHP). This is important, because Na^+^,K^+^-ATPase operation during clearance of excess K^+^ from the extracellular space may create extracellular hypertonicity, as demonstrated after neuronal activity by Dietzel et al. (1982, 1989). The neuronal effect of hyperosmolarity must be mediated via astrocytes, since acute hyperosmotic stimulus-induced Fos expression in neurons depends on initial activation of astrocytes (Yuan et al., 2010). *Reduction* of sAHP by β-adrenergic activity [and in some situations by activation of other transmitter receptors (Araneda and Andrade, 1991; Fisahn et al., 2005)] is thought to be immensely important for memory-enhancing effects, especially of noradrenaline and isoproterenol (Oh et al., 2009; Zaitsev and Anwyl, 2012). During the isoproterenol-induced regulatory volume increase glycogenolysis again fulfills Na^+^,K^+^-ATPase's signaling requirements and thus enables the NKCC1 cotransporter to create an undershoot in extracellular K^+^ concentration *and* to enhance regulatory volume increase. Since isoproterenol-mediated stimulation of Na^+^,K^+^-ATPase activity is prevented at elevated K^+^, sAHP can only be activated after termination of extracellular K^+^ clearance.

More than 35 years ago Gibbs and Ng (1977) demonstrated inhibition of memory when Na^+^,K^+^-ATPase activity is prevented by conventional inhibitory concentrations of ouabain (Figure 2). The study also showed that adrenaline could counteract the inhibition. Additional papers by the Gibbs group have further demonstrated temporal and hemispheric details of the amnestic effects of ouabain administration (Watts and Mark, 1971; Robertson et al., 1978; Bell and Gibbs, 1979; Tucker and Gibbs, 1979; Crowe et al., 1989; Gibbs et al., 2003). The β_2_-adrenergic agonist zinterol was not available at that time, but recent experiments by Marie E. Gibbs have shown that zinterol also counteracts the inhibitory effect of ouabain (personal communication). This observation provides *in vivo* support for the importance of β-adrenergic interaction with Na^+^,K^+^-ATPase function during learning, with stimulation of regulatory volume increase and concomitant reduction of sAHP being the most likely explanation for the rescue of memory. In this context, it should be noted that Gibbs and Ng (1976) suggested 37 years ago that “the possible hyperpolarization of membrane potential associated with sodium pump activity may serve to mark the labile memory trace” until changes into more permanent memory take place. More recent studies (Disterhoft et al., 1986; Oh et al., 2009) have shown that in many types of learning the opposite occurs: after learning sAHP becomes reduced even in the absence of transmitter activity, so the neuron becomes more easily recruited, and transmitter application has no further effect. Nevertheless, Gibbs and Ng (1976) remain the first to have contemplated an association between neuronal afterhyperpolarization and learning.

**Figure 2:**
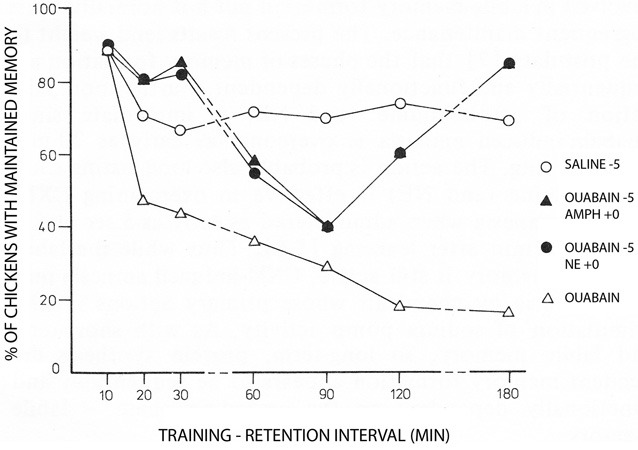
**Day-old chicks were during training exposed to a red bead tainted with the aversively tasting drug anthranilate and to an untainted blue bead.** The Figure shows the percentage of trained chickens, which have learned to avoid the red color, which at the memory test was untainted. Under control conditions ~70% remember. This number was rapidly reduced by administration of ouabain 5 min before training (with saline added to the controls at the same time) to reach <20% after 2–3 h. However, the amnesic effect of ouabain was initially, and after a transient dip in memory, also at later times prevented by noradrenaline (NE) given at the time of learning (summarized on the Figure as ‘ouabain −5, NE +0). Amphetamine administered using the same time schedule, had a similar effect, probably due to noradrenaline release. Each point represents the average of measurements in 18–20 birds. For further details, see Gibbs and Ng (1977), from where the Figure is reproduced, with permission. For later procedural improvements see, e.g., Gibbs et al. (2008).

An ability of either elevated K^+^ concentrations (Kaufman and Driscoll, 1993) or either noradrenaline or clonidine, an α_2_-adrenergic receptor agonist (Chen and Hertz, 1999), to stimulate rate of pyruvate carboxylation might suggest that signaling events akin to those involved in regulation of K^+^ uptake also might be involved during activation of pyruvate carboxylase. As discussed above, α_2_-adrenergic receptor stimulates glycogen synthesis. The studies on pyruvate carboxylation by Chen and Hertz (1999) were carried out in glucose-free medium, so glycogen may have become absent, rendering it impossible to know if β-adrenergic stimulation would otherwise have been effective.

In conclusion, glycogen's unaltered degradation under control conditions, and the immediate response to a slight increase in extracellular K^+^ concentrations or transmitter release may explain its importance for glutamate formation, cellular K^+^ homeostasis and probably also for reduction of the excitation-inhibitory sAHP. Need of glycogenolysis for glutamate synthesis by astrocytes, contributions by astrocytes to clearance of extracellular K^+^ increase following neuronal activity, and astrocytic participation in sAHP reduction all seem to contribute to glycogen's unique, pluralistic role in *integrative* establishment of memory. Both *in vivo* studies and culture studies have concurred in the importance of glycogenolysis, and thus of astrocytic metabolism, for glutamate production. Its role in K^+^ clearance from the extracellular space following neuronal activity has not yet been *directly* established *in vivo*. However, there is abundant supportive *in vivo* evidence (see Xu et al., 2013). Possible importance of glycogenolysis for reduction of sAHP could be investigated *in vivo*, as could roles of glycogenolysis for interactions between learning and sAHP reduction. The robust evidence for glycogen's importance in learning shown by Duran et al. (2013), together with information reviewed in this perspective suggest that such studies could make essential contributions toward understanding “how multiple diverse functions are integrated to produce complex behaviors,” such as learning.

## Conflict of interest statement

The authors declare that the research was conducted in the absence of any commercial or financial relationships that could be construed as a potential conflict of interest.
